# Photonics probing of pup brain tissue and molecular-specific nuclear nanostructure alterations due to fetal alcoholism via light scattering/localization approaches

**DOI:** 10.1117/1.JBO.27.7.076002

**Published:** 2022-07-11

**Authors:** Prakash Adhikari, Pradeep K. Shukla, Fatemah Alharthi, Shiva Bhandari, Avtar S. Meena, Radhakrishna Rao, Prabhakar Pradhan

**Affiliations:** a Mississippi State University, Department of Physics and Astronomy, Mississippi State, Mississippi, United States; b University of Tennessee Health Science Center, Department of Physiology, Memphis, Tennessee, United States

**Keywords:** light scattering, light localization, mesoscopic physics, partial wave spectroscopy, inverse participation ratio, structural disorder, fetal alcoholism

## Abstract

**Significance:**

Light is a good probe for studying the nanoscale-level structural or molecular-specific structural properties of brain cells/tissue due to stress, alcohol, or any other abnormalities. Chronic alcoholism during pregnancy, i.e., fetal alcoholism, being teratogenic, results in fetal alcohol syndrome, and other neurological disorders. Understanding the nano-to-submicron scale spatial structural properties of pup brain cells/tissues using light/photonic probes could provide a plethora of information in understanding the effects of fetal alcoholism.

**Aim:**

Using both light scattering and light localization techniques to probe alterations in nano- to-submicron scale mass density or refractive index fluctuations in brain cells/tissues of mice pups, exposed to fetal alcoholism.

**Approach:**

We use the mesoscopic physics-based dual spectroscopic imaging techniques, partial wave spectroscopy (PWS) and molecular-specific inverse participation ratio (IPR) using confocal imaging, to quantify structural alterations in brain tissues and chromatin/histone in brain cells, respectively, in 60 days postnatal mice pup brain, exposed to fetal alcoholism.

**Results:**

The finer focusing PWS analysis on tissues shows an increase in the degree of structural disorder strength in the pup brain tissues. Furthermore, results of the molecular-specific light localization IPR technique show an increase in the degree of spatial molecular mass density structural disorder in DNA and a decrease in the degree in histone.

**Conclusions:**

In particular, we characterize the spatial pup brain structures from the molecular to tissue levels and address the plausible reasons for such as mass density fluctuations in fetal alcoholism.

## 1 Introduction

Light is a probe used to detect the structural properties of cells/tissues. Nano-to-submicron scale intracellular structural alterations that happen inside the cells, mainly in macromolecules, such as DNA, RNA, lipids, etc. due to disease, chronic alcoholism, or any other abnormalities. It is now known that structural changes in the macromolecules of cells result in fundamental mass density changes in the cell/tissue structure. A mesoscopic physics-based imaging technique, partial wave spectroscopy (PWS), has the sensitivity to discern nanoscale structural changes and can distinguish the mass density fluctuations in terms of refractive index fluctuations due to cancer, stress, or chronic alcoholism in cells/tissues.^1^^–^^5^ Besides the structural changes in tissues, there are different levels of molecular-specific nano-to-submicron scale structural alterations that happen in the cell nuclei, which are in some way related to disease or abnormalities. The molecular-specific photonic localization technique using confocal imaging, named inverse participation ratio (IPR) technique, is a highly sensitive technique recently used to probe and quantify the structural alterations in cells/tissues due to cancer, alcohol, or other drugs^6^^–^^9^ including structural changes in the brain cells.^10^

Alcohol consumption has been a part of human culture since the beginning of human civilization; however, chronic alcoholism is responsible for several health issues in the present day. Many major health problems reported are directly or indirectly associated with alcohol consumption. The physical change due to alcohol in the cells/tissues of a particular organelle depends on various factors such as age, sex, amount consumed, the concentration of alcohol, etc. Existing literature claims that alcohol affects all the major organs of the body, such as the liver, heart, pancreas, kidney, brain, etc. The immune system is degraded by chronic alcohol consumption, and the body is prone to disease thereafter.^11^ Therefore, alcohol consumption in the short term is a major cause of increased nausea, and in the long run, results in death.^12^ Researchers have shown that a complex and multidimensional relationship occurs between alcohol consumption and health consequences.^13^ Chronic alcoholism is, therefore, an essential factor responsible for different health concerns. The Center for Disease Control and Prevention estimates a death toll of 95,000 annually from excessive alcohol use, and alcohol is one of the leading preventable causes of death in the United States. The number of deaths due to chronic alcoholism is increasing year by year.

Alcohol has been identified as a carcinogen and is responsible for most cancers.^14^ Most of the research on alcohol has shown that it has more effects on women than on men.^15^ Chronic alcoholism of a mother during pregnancy, the cause of fetal alcoholism, is one of the major risk factors of mental retardation in children born to such mothers in the United States and worldwide.^16^ Fetal alcoholism leads to several critical health issues such as miscarriage, stillbirth, and different disabilities known as fetal alcohol spectrum disorders.^17^ Fetal alcohol syndrome (FAS) and alcohol-related neurodevelopmental disorder are severe outcomes of fetal alcoholism that are linked with notable cognitive and behavioral deficits to a newborn child.^18^ A child born from every 13 mothers who consumed alcohol during pregnancy is found to have fetal alcohol disorder syndrome (FADS). The number of infants born with FADS is estimated to be 630,000 annually worldwide, and this number is increasing every year.^19^ This early preventable issue is tragic as it is a leading cause of intellectual disabilities, congenital disabilities, and developmental disorders.^20^ These effects demand early and accurate quantification of structural abnormalities due to fetal alcoholism in pup brain cells/tissues.

Several factors are responsible for the complications in analyzing the effects of alcohol exposure on fetus brain development. The condition of changing the blood alcohol concentration in the fetus plays a vital role in influencing the occurrence and harshness of alcohol-induced developmental brain-injuries.^21^ The alcohol consumed by the pregnant mother passes through the placenta to the growing baby in the womb who consumes alcohol in the same amount as the mother. The baby is unable to metabolize the alcohol through its liver or any other organs in the fetus. Therefore, alcohol, being teratogenic, interferes with the healthy growth of vital organs such as the brain and results in brain damage and other congenital disabilities. These brain cells/tissues are initially affected at the nano- to submicron-levels, which look similar to normal cells/tissues and remain unpredicted by current histological procedures. At this point, the structural alterations in alcoholic mothers’ pups’ brain tissues and cell nuclei are not well studied or understood. Therefore, we report the study of mesoscopic physics-based dual spectroscopic imaging techniques, the PWS and IPR, which can provide a plethora of information on the structural changes at submicron-scale levels in tissues and nuclei of brains of pups exposed to fetal alcoholism. First, we probe the nanoscale to submicron-scale refractive index fluctuations in thin brain tissue sections using the PWS technique to quantify the degree of structural disorder strength ($L_{d\text{-}\mathrm{PWS}}$) at the tissue level. Then, we examine the spatial structural disorder changes ($L_{d\text{-}\mathrm{IPR}}$) in the molecular mass density of nuclear components, DNA (DAPI stained), and histone protein (H3K27me3 antibody stained), using the IPR technique via confocal imaging (confocal-IPR).

The rationale for using PWS and IPR techniques are as follows: (i) the PWS technique measures the degree of structural disorder parameter $L_{d\text{-}\mathrm{PWS}}=dn^{2}\times lc$ from optical backscattering experiment and (ii) the IPR method measures another structural disorder parameter $L_{d\text{-}\mathrm{IPR}}=dn\times lc$, where *dn* is the standard deviation (std) of the onsite refractive index fluctuation and lc is its spatial correlation decay length of the refractive index fluctuation. Therefore, this method is a dual-spectroscopic probing of the sample for two different parameters related to the degree of structural disorder or disorder strength. In both these techniques, the parameters (or biomarkers) can be expressed as a function of *dn* and lc, in different ways. Further rationale for using the confocal-IPR method is that pure confocal images provide only the mass density figure of a particular molecular spatial mass density; however, the confocal-IPR method provides its nano- to submicron-scale molecular-specific light localization properties, therefore, the IPR method is very sensitive for nano- to submicron-scale structural changes.

The details of the PWS study of brain tissue samples and the confocal-IPR study of DNA and histones of brain cell nuclei and obtained results, their significance, and applications are described as follows.

## 2 Method

### 2.1 Brain Cell and Tissue Samples Preparation

All animal experiments were performed following the protocol approved by the University of Tennessee Health Science Center (UTHSC) Institutional Animal Care and Use Committee (IACUC). Mice were housed in groups of 3 to 5 per cage, segregated by sex, in a room on a 12 h/12 h light/dark cycle (lights on at 8:00 AM and off at 8:00 PM) maintained at 22±2°C. Pregnant mice for 6 weeks were fed with ethanol (0% 2 days, 1% 2 days, 2% 2 days, 4% 1 week, and 5% 1 week) in Lieber-DeCarli liquid diet (EF). The control group was pair fed with an isocaloric diet. Brain sections from the control or pair fed (PF) and ethanol fed (EF) offspring were collected at 60-days postnatal age and cryofixed for fluorescence staining using confocal microscopy. While the offspring of PF and EF mice were excised and the brain tissue sections from the frontal cortex region were cryofixed and sectioned into a thickness of 10  μm using microtome for the PWS and IPR analyses.

### 2.2 Partial Wave Spectroscopy for Structural Disorder Detection

#### 2.2.1 PWS instrumentation

PWS is a recently introduced highly sensitive imaging technique that combines mesoscopic physics-based analysis with spectroscopic images to detect the nanoscale-scale structural alterations in cells/tissue. A detailed experimental explanation of the PWS instrumentation is presented in the previous work.^3^^,^^5^^,^^22^^,^^23^ In brief, a stable broadband low coherence white light source, Xenon Lamp (150cW) is passed through the 4f combination of lenses and gets collimated. With a right-angle prism, the collimated beam is reflected toward the samples through the objective lens (40X, NA=0.65, power ∼3  mW at the sample size ∼1  mm, Newport). A highly sensitive XYZ motorized stage (X–Y axis 40 nm and Z-axis 100 nm, Zebar Technologies) is used to focus the collimated beam into the sample within the working distance of the objective, and this finer focusing PWS. The resulting spectroscopic backscattered signal is projected toward the CCD camera filtered with the liquid crystal tunable filter (LCTF, Kurios), which has a resolution of 1 nm. Finally, for every wavelength in the visible range (450 to 700 nm) of light backscattered signals are recorded in the CCD camera. The CCD camera and LCTF are triggered by the LCTF controller and signals at every wavelength are recorded using the CCD.

#### 2.2.2 Calculation of disorder strength in the PWS ($L_{d\text{-}\mathrm{PWS}}$)

In the PWS technique, the sample is virtually divided into a collection of parallel channels each with diffraction-limited transverse size, to detect the backscattered waves propagating along quasi one-dimensional (1D) trajectories within these channels. The backscattered signals at every wavelength (λ) and each spatial pixel position (x,y) are recorded in the CCD camera as R(x,y,λ), and then the statistical properties of the nanoarchitecture of tissue are quantified by analyzing the fluctuating part of the recorded backscattered intensities. The collected backscattered signals are applied with a Butterworth filter and suitable low order polynomial to remove the noise. Then, the degree of structural disorder strength, $L_{d\text{-}\mathrm{PWS}}$, is calculated from the *rms* value of the extracted intensities ${\left\langle R(k)\right\rangle }_{\mathrm{rms}}$ and the wave vector autocorrelation decay of the reflection intensity ratio, C(Δk). At the spatial pixel position (x,y), the degree of structural disorder strength, $L_{d}$ is given as^1^^,^^3^^,^^22^^,^^24^^,^^25^

$$L_{d\text{-}\mathrm{PWS}}={\frac{Bn_{0}^{2}{\left\langle R\right\rangle }_{\mathrm{rms}}}{2k^{2}}\frac{{\left(\Delta k\right)}^{2}}{-\ln(C(\Delta k))}|}_{\Delta k\to 0},$$ (1)

where B is the normalization constant, $n_{0}$ is the average refractive index of the cells/tissue, and k is the wavenumber (k=2π/λ). This disorder strength can be further simplified and expressed as the product of the variance and the spatial correlation length of the refractive index fluctuations, $L_{d\text{-}\mathrm{PWS}}=\langle \Delta n^{2}\rangle l_{c}$.

### 2.3 Confocal Microscopy for DNA/Histone Spatial Molecular Mass Density Structural Disorder Detection

#### 2.3.1 DNA and histone staining of nuclei in tissue samples for molecular-specific structural disorders

The immunofluorescence staining was performed in cell nuclei of brain sections with two types of molecular mass densities: DNA and histone.

(1) DNA staining: Nuclear DNA treated with DAPI, a nuclear dye that binds to mainly DNA and in turn, chromatin. Tissue sections are washed three times for 5 min in phosphate buffered saline (PBS) and mounted on a glass slide using Prolong Diamond antifade mountant containing DAPI. The DAPI present in the mountant is a DNA binding dye that enables the visualization of the nuclei by fluorescence microscopy.
(2) Histone staining: Immunofluorescence staining of histone was performed using the H3K27me3 antibody. Cryosections (10  μm thick) were fixed in a one-to-one mixture of acetone and methanol at −20°C for 2 min and rehydrated in PBS. PBS is a mixture of 137 mM sodium chloride, 2.7 mM potassium chloride, 10 mM disodium hydrogen phosphate, and 1.8 mM potassium dihydrogen phosphate. Sections were permeabilized with 0.2% Triton X-100 in PBS for 10 min and blocked in 4% non-fat milk in Triton–Tris buffer (150 mM sodium chloride containing 10% Tween-20 and 20 mM Tris, pH 7.4). It was then incubated for 1 h with the primary antibodies (rabbit polyclonal anti-H3k27me3), followed by incubation for 1 h with secondary antibodies (cy3-conjugated anti-rabbit IgG antibodies).

#### 2.3.2 Confocal imaging

Confocal imaging was performed on DAPI and H3K27me3 antibody-stained nuclei of cells in their native states in tissues, collected from the frontal cortex region of a mouse model. The fluorescence was examined using a Zeiss710 confocal microscope (Carl Zeiss Microscopy, Jena, Germany), and images from x–y sections (1 mm) were collected using the provided software. Images were stacked using the software ImageJ (NIH, Bethesda, Maryland), and processed by Adobe Photoshop (Adobe Systems Inc., San Jose, California). All images for DNA and histone tissue samples from different groups were collected and processed under identical conditions. The images obtained were categorized into four groups: (i) PF-DAPI-stained brain cells for DNA structure for control, (ii) EF-DAPI-stained brain cells for DNA structures of EF mother, (iii) PF-H3K27me3-stained brain cells for histone structure for control, and (iv) EF-H3K27me3-stained brain cells for histone structures of EF mother. For confocal imaging, excitations/emissions: 358  nm/461  nm for DAPI and 680  nm/570  nm for H3K27me3, respectively.

#### 2.3.3 Inverse participation ratio and molecular-specific structural disorder analysis

The method of confocal imaging and quantification of light localization properties of the biological system has been described previously and details are given elsewhere.^6^^,^^26^ The fluorescence emitted by the sample molecules from a small finite volume or voxel, i.e., (dV=dxdydz) around the excitation center is collected by the photodetector. The confocal image intensity I(r) is found to be Ref. 26, where I(r) is the pixel intensity of the confocal image at position r(x,y), and ρ is the density of the molecules at a small volume dV. It has been shown that the local refractive index of a cell is proportional to its local mass density,^26^^–^^28^ i.e., $n(r)=n_{o}+\Delta n(r)$. So, a representative refractive index matrix of the fluorescence molecular mass density variation is constructed using the pixel intensity values, with the optical potential ε(x,y) defined as

$$\varepsilon (r)=\frac{\Delta n(x,y)}{n_{0}}\propto \frac{dI(x,y)}{\left\langle I\right\rangle },$$ (2)

where $n_{0}$ and Δn(x,y) denote the average refractive index of the fluorescent molecules and their fluctuation at (x,y) position (area dxdy and thickness dz), respectively. ⟨I⟩ is the average intensity of the confocal images and dI(x,y) is the intensity fluctuation at (x,y) pixel position of the confocal image. From this, an optical lattice is a representation of the spatial refractive index fluctuations of the fluorescent molecules inside the sample.^29^ The Anderson Tight Binding Model (TBM) is well studied in condensed matter physics for describing the disorder properties of optical systems of any geometry and disorder.^30^ If we consider one optical state per lattice site, with inter lattice site hopping restricted to the nearest neighbors only, the tight-binding Hamiltonian can be written as

$$H=\underset{i}{\sum }\varepsilon_{i}|i\rangle \langle i|+t\underset{\left\langle ij\right\rangle }{\sum }(|i\rangle \langle j|+|j\rangle \langle i|),$$ (3)

where $\varepsilon_{i}$ is the optical lattice potential corresponding to the i’th lattice site, j’th is the nearest neighbor of the i’th lattice site, and t is the interlattice site hopping strength. The average IPR value for a lattice system is calculated from the eigenfunction of the above Hamiltonian as

$$\langle \mathrm{IPR}(L)\rangle_{L\times L}=\frac{1}{N}\overset{N}{\underset{i=1}{\sum }}\int_{0}^{L}\int_{0}^{L}E_{i}^{4}(x,y)dx\;dy,$$ (4)

where $E_{i}$ is the i’th eigenfunction of the Hamiltonian of an optical lattice of size L×L, having N lattice points. Two parameters can specify the disorder in heterogeneous light transparent media such as biological systems, the variance of the refractive index fluctuations Δn and its spatial fluctuation correlation length $l_{c}$ and can be expressed as disorder strength, $L_{d}$:

$$L_{d}=\langle \Delta n\rangle \times l_{c},$$ (5)

and the average value of ${\left\langle \mathrm{IPR}(L)\right\rangle }_{L\times L}$ represents the measure of the disorder strength, so the $\left\langle {\left\langle \mathrm{IPR}(L)\right\rangle }_{L\times L}\right\rangle$ and disorder strength can be written as^6^^,^^26^

$$L_{d-\mathrm{IPR}}\equiv \langle \mathrm{IPR}\rangle =\langle {\left\langle \mathrm{IPR}(L)\right\rangle }_{L\times L}\rangle =\langle \Delta n\rangle \times l_{c.}$$ (6)

## 3 Results

### 3.1 Partial Wave Spectroscopy Study of Brain Tissues

The light scattering, PWS technique and molecular-specific light localization, confocal-IPR technique were used to detect the nano-to-submicron scale structural alterations in the mice pup brain tissue and nuclei due to fetal alcoholism. For this, the PWS analysis was performed in the paraffin-embedded 10-μm thick on the PF and EF mice pup brain tissue sections, and the degree of structural disorder strength, $L_{d\text{-}\mathrm{PWS}}$, was calculated as mentioned in Sec. 2.2. These pup brain cells/tissues were collected at postnatal day 60 to quantify their structural properties due to exposition to fetal alcoholism. Then, using the confocal-IPR analysis, the average IPR, ⟨IPR⟩ value was calculated at sample length L=0.8  μm to study the effect of alcohol in EF mice pup brain cell nuclei, especially in the DNA and histone for all the categories as explained in Sec. 2.3. Here, the IPR study was performed and compared between (i) PF DAPI-stained brain cells and EF DAPI-stained brain cells and (ii) PF H3K27me3-stained brain cells and EF H3K27me3-stained brain cells.

**Fig. 1:**
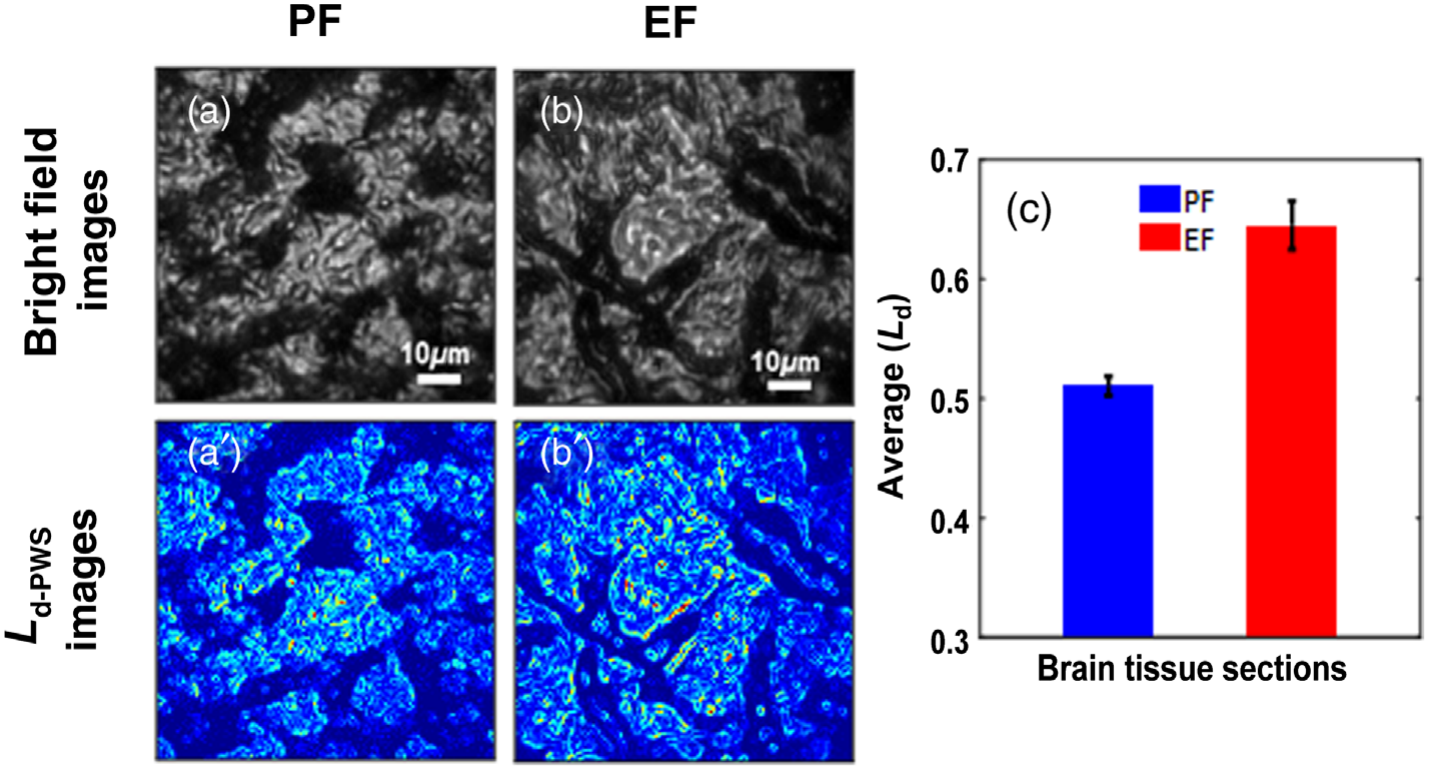
Brain tissue structural disorder using PWS: (a)–(b) the bright field images of PF and EF mice pup brain tissue while (a′)–(b′) their respective $L_{d\text{-}\mathrm{PWS}}$ images, which are distinct than the bright field images. (c) The EF mice pup’s brain tissues have a higher degree of the average disorder strength, $L_{d\text{-}\mathrm{PWS}}$ than PF pups. The average $L_{d\text{-}\mathrm{PWS}}$ of EF mice pup brain tissue increases by 26% in reference to the PF pups. (Student t-test, P-value<0.05, PF mice (n=5) and EF mice (n=5), 3 to 5 samples per mouse).

Figures 1(a)–1(b) and 1(a′)–1(b′) represent the bright field images and $L_{d\text{-}\mathrm{PWS}}$ images, respectively, of PF and EF mice pup thin brain tissue from frontal cortex sections. Based on quasi-1D approximation in PWS, the samples were virtually divided into several parallel channels, and the backscattered signals propagating along 1D trajectories were collected to calculate the degree of structural disorder strength ($L_{d\text{-}\mathrm{PWS}}$) at every pixel position. This disorder strength is calculated in terms of the refractive index fluctuations is represented in a 2D color map of the $L_{d\text{-}\mathrm{PWS}}$ image in Fig. 1(c), where the red color represents the higher refractive index fluctuations present in the tissue section averaged along the z-axis. Although the bright field images look similar, the $L_{d}$ images show that the EF pup brain tissue structure has more red spots indicating a higher degree of refractive index fluctuations or the disorder strength than the PF pup. This is due to the alcohol effect in the fetus of alcoholic mothers’ pups. Further, the average disorder strength ($L_{d\text{-}\mathrm{PWS}}$) was calculated and represented in Fig. 1(c). The PWS quantification shows that the average degree of disorder strength of EF mice pup brain tissue increases significantly by 26% compared to the PF pup. This increase in the degree of disorder strength of EF pup brain tissue structure is due to the adverse effects of alcohol in the brain of pups, as a result of alcohol consumption by the mother during her pregnancy. Since alcohol consumed during pregnancy is equally consumed by the fetus may result in different neurological abnormalities in the newborn. It is found that alcohol affects the different brain cells, such as astrocytes, microglia, etc. including cell components such as chromatin, histone, etc. which are initially at the nanoscale level eventually leading to brain malfunctioning. FAS and alcohol-related neurodevelopmental disorders in newborn infants are the long-term outcomes of fetal alcoholism. This quantitative approach to the measurement of such structural alterations in the brain tissue due to fetal alcohol exposure (FAE) using the PWS technique provides a better understanding of the structural properties at the earliest stages of fetal alcoholism to employ better treatment modalities.

### 3.2 Confocal-IPR Study of: (i) DNA Molecular-Specific DAPI Staining and (ii) Histone Molecular-Specific H3K27me3 Staining

**Fig. 2:**
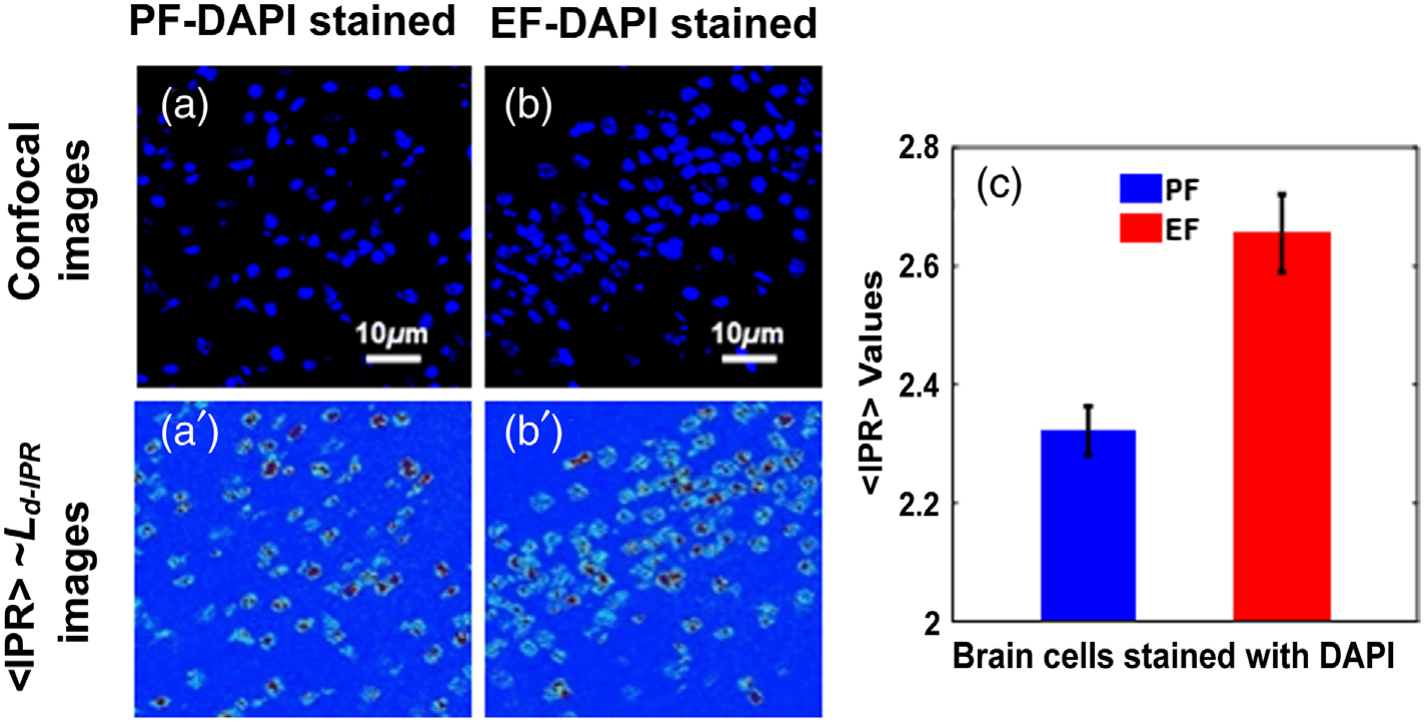
DNA molecular-specific structural disorder by confocal-IPR. (a)–(b) The confocal images of the PF and EF DAPI-stained pup brain cells while (a′)–(b′) are their respective IPR images. (c) The $\langle \mathrm{IPR}\rangle ∼L_{d\text{-}\mathrm{IPR}}$ values for PF DAPI-stained and EF DAPI-stained pup brain cells at sample length L=0.8  μm are presented. The ⟨IPR⟩ value for EF DAPI-stained brain cells was found to be higher compared to that of PF DAPI-stained brain cells. As DAPI binds with DNA molecules, the above bar plots show that EF pups have more structural disorder in their brain cell nuclei leading to different kinds of abnormalities. The percentage difference between the ⟨IPR⟩ values of these two groups is 14%. (Student t-test, P-value<0.05, PF mice (n=5) and EF mice (n=5), 3 to 5 samples per mouse).

**Fig. 3:**
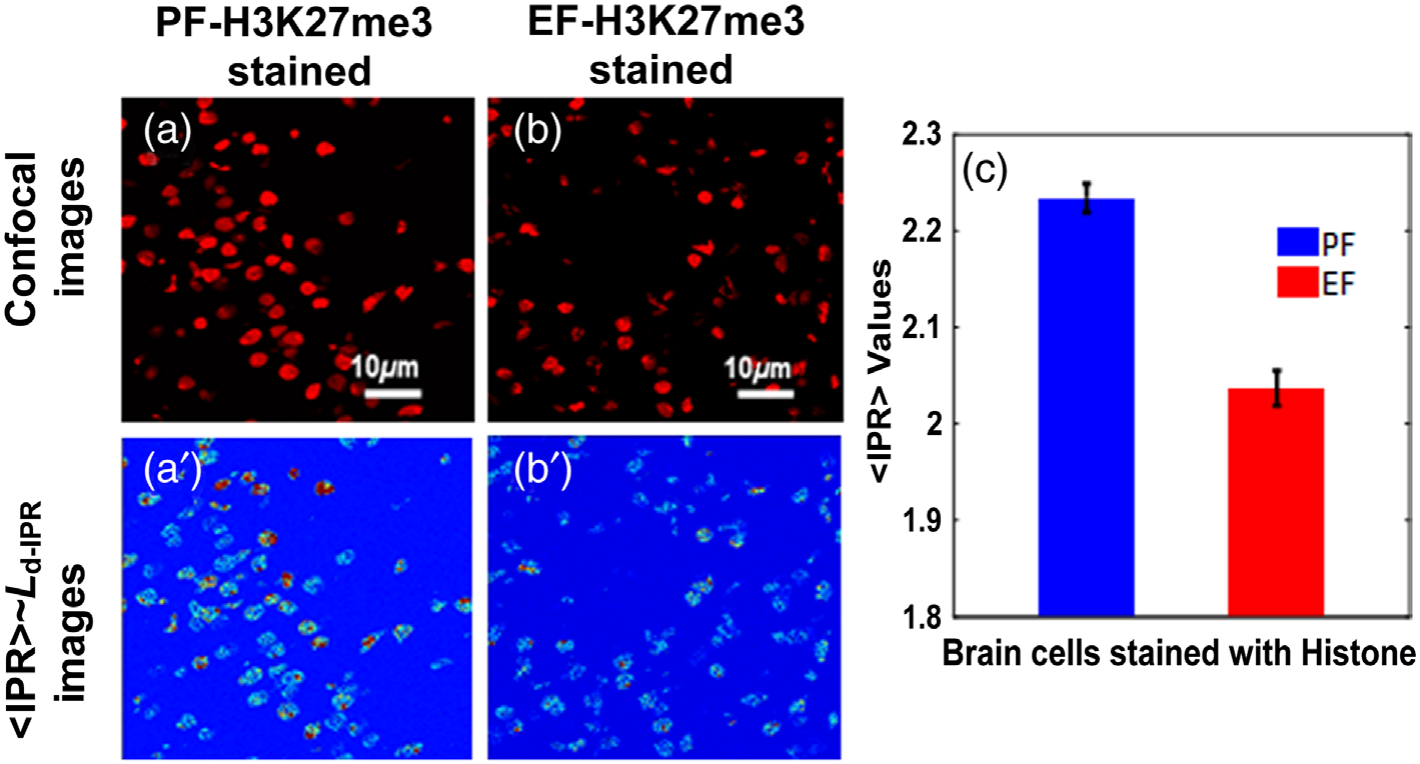
Histone molecular-specific structural disorder by confocal-IPR: (a)–(b) the confocal images of the PF and EF H3K27me3-stained pup brain cells targeting histone, and (a′)–(b′) represent their respective ⟨IPR⟩ images. (c) The ⟨IPR⟩ values or the degree of structural disorder at sample length L=0.8  μm for PF H3K27me3-stained and EF H3K27me3-stained pup brain cells are presented. The ⟨IPR⟩ value for PF H3K27me3-stained brain cells was found to be lower compared to that of the PF H3K27me3-stained brain cells. The percentage difference of the ⟨IPR⟩ values for structural disorder between these two groups is 10%. (Student t-test, P-value<0.05 PF mice (n=5) and EF mice (n=5), 3 to 5 samples per mouse.)

Figures 2(a)–2(b) show the DAPI-stained mice pup brain cells for PF and EF, respectively, and 2(a′)–2(b′) are the corresponding ⟨IPR⟩ images of these cells. DAPI staining mainly targets DNA molecules in the brain cell nuclei. Figure 2(c) shows the bar graph comparison of ensemble ⟨IPR⟩ values at the sample length L=0.8  μm of PF and EF DAPI-stained pup brain molecules. The result shows the higher ⟨IPR⟩ value or disorder strength ($L_{d\text{-}\mathrm{IPR}}$) for EF pup brain cells, indicating higher structural disorder than the PF pup brain cells. This result suggests that the ⟨IPR⟩ value of DNA increased due to fetal alcoholism. This is due to an increase in the mean mass density fluctuation in DNA/chromatin of alcoholic mice pup brain cell nuclei. Figures 3(a)–3(b) represent the H3K27me3 antibody-stained pup brain cells for the PF and EF, respectively, while 3(a′)–3(b′) are their corresponding ⟨IPR⟩ images. H3K27me3 antibody staining mainly targets the histone molecules in the brain cell nuclei. Figure 3(c) shows the bar graph comparison of the ensemble ⟨IPR⟩ values for PF H3K27me3-stained and EF H3K27me3-stained pup brain cells. The graphs show histone structures in pup brain cells exposed to fetal alcoholism has less ⟨IPR⟩ or structural disorder relative to the PF pup. This, the opposite trend of the nuclear DNA molecular structure, shows an interesting result as an adverse effect of fetal alcoholism.

The ⟨IPR⟩ values at the sample length L=0.8  μm targeting DNA molecules of EF pup brain cells nuclei are found to be higher than the PF pup. This ⟨IPR⟩ value is correlated with the structural abnormalities, which indicates that the spatial mass distributions or fluctuations of DNA molecules in an EF pup’s brain cells are higher. The introduction of alcohol during pregnancy is consumed by the fetus and results in spatial variation in the components of DNA at the nanoscale level and hence is responsible for the changes in genetics and results in different fetal alcoholic syndromes.

However, the ⟨IPR⟩ value of EF H3K27me3 antibody-stained brain cells targeting histone is found to be less than the ⟨IPR⟩ value of PF H3K27me3-stained pup brain cells at sample length L=0.8  μm. This suggests that the mass density fluctuations decrease in EF H3K27me3-stained pup’s stained brain cells compared to the PF pup suggesting some effect of alcohol in histone molecular mass density. And, this decrease in mass density fluctuations in the histone proteins of brain cells due to fetal alcoholic exposition concludes suppression in certain gene expression resulting in FAS.

### 3.3 Quantitative RT-PCR Study for Measure the DNA Damage to Support the Structural Disorder

#### 3.3.1 Quantitative RT-PCR method

Here, we quantify the amount of DNA damage using a quantitative RT-PCR study. Total RNA (1.5  μg) was used for the generation of cDNAs using the ThermoScript RT-PCR system for first strand synthesis (Invitrogen, Carlsbad, California). Quantitative PCR (qPCR) reactions were performed using cDNA mix (cDNA corresponding to 35 ng RNA) with 300 nmol of primers in a final volume of 25  μL of ×2 concentrated RT2 real-time SYBR Green/ROX master mix (Qiagen, Venlo, the Netherlands) in an Applied Biosystems 7300 real-time PCR instrument (Norwalk, Connecticut). The cycle parameters were 50°C for 2 min, one denaturation step at 95°C for 10 min, and 40 cycles of denaturation at 95°C for 10 s followed by annealing and elongation at 60°C. Relative gene expression of each transcript was normalized to GAPDH using the ΔΔCt method.^31^ Sequences of primers used for qPCR are provided in Table 1.

**Table 1:** Sequences of primers used for qPCR experiment.

**γH2AX**
Forward primer: AACGACGAGGAGCTCAACAAGC
Reverse primer: TGGCGCTGCTCTTCTTGGGCA
**GAPDH**
Forward primer: CTGCACCACCAACTGCTTAG
Reverse primer: GGGCCATCCACAGTCTTCT

**Fig. 4:**
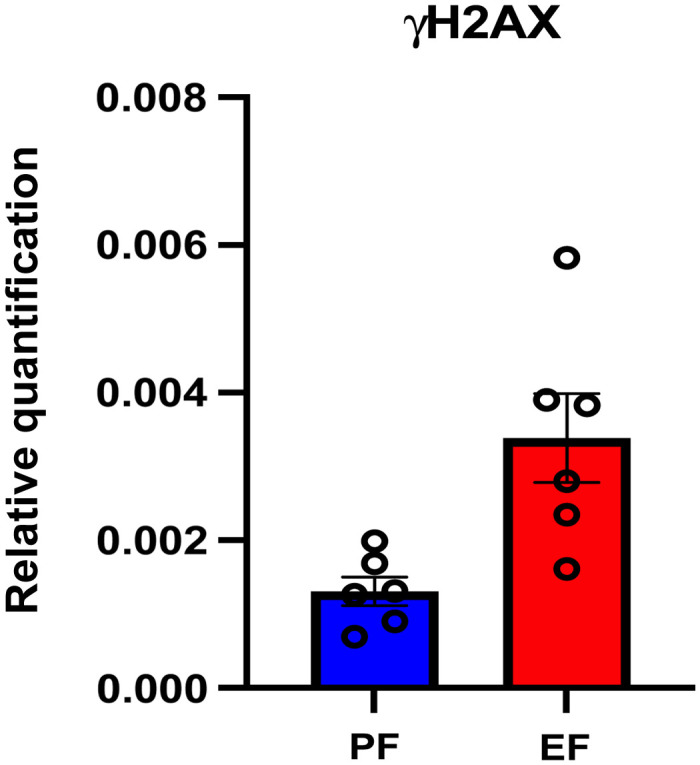
FAE litters showed DNA damage marker gene (γH2AX) expression changes in the frontal cortex. Relative quantification measured by real-time RT-PCR indicates an increase in γH2AX transcript levels in the frontal cortex of adult litters of EtOH consuming mother.^31^ Asterisk indicates the value that is significantly different from corresponding control value (Student’s t-test, P-value<0.05, PF mice (n=5) and EF mice (n=5), 3 to 5 samples per mouse).

Expression of DNA damage marker gene was analyzed by measuring the specific mRNA levels in the frontal cortex. Levels of mRNA for γH2AX were significantly increased in litters of EF dams compared to those in the litters of PF dams at postnatal day 60, as shown in Fig. 4.

## 4 Conclusions and Discussions

The effects of chronic alcoholism during pregnancy or fetal alcoholism on pups’ brains were studied for the first time using the mesoscopic physics-based dual spectroscopic approach, the PWS, and confocal-IPR techniques, in a mouse model. Based on the PWS technique, we quantified the mass density or refractive index fluctuations at the nano-to-submicron levels expressed as the degree of structural disorder strength ($L_{d\text{-}\mathrm{PWS}}$) in PF, and EF mice pup brains. The PWS result shows an increase in the degree of structural disorder strength of brain tissue in mice pups exposed to fetal alcoholism relative to PF (control fed) pup, which indicates alcohol is consumed by the fetus and has an adverse effect on the brain. This study was further followed by the confocal-IPR technique, to probe the spatial structural disorder properties of the molecular-specific mass density of two types of molecules in cells: (i) DNA and (ii) histone. The results show the degree of molecular-specific structural disorder, $L_{d\text{-}\mathrm{IPR}}$, for DNA increases while that of histone decreases. In addition, this variation in molecular-specific structural changes in DNA and histone may be due to DNA methylation.^3^ As discussed in Sec. 1, the rationale for using the IPR light localization method is to quantify the structural disorder property of the sample in quantitative and effective way, and this can be seen in the relative $L_{d\text{-}\mathrm{IPR}}\text{ color}$ maps compare to relative confocal maps, which is quite different from relative IPR confocal maps, between control and treated. The nanoscale changes in pup brain tissue or cellular components from the alcoholic mice are due to the structural alterations in some specific components of the tissue/nuclei that result in mass density variation or refractive index variation. This change in spatial structural disorder from tissue to the molecular mass densities of DNA and histones may result in neurological abnormalities which are initially at the nano-to-submicron scale level in the fetus due to fetal alcoholism. And eventually, these brain structural abnormalities may result in FAS and other alcohol related neurodevelopmental disorders in infants as long-term effects of fetal alcoholism.

### 4.1 Quantitative RT-PCR Experiment Supports an Increase in the Structural Disorder by DNA Damage

Specific mRNA levels in the frontal cortex were quantified by measuring the expression of the DNA damage marker gene. Results show that the levels of mRNA for γH2AX were significantly increased in litters of EF dams compared to those in the litters of PF dams at postnatal day 60. It is known that molecular-level damage increases the spatial disorder in a system. Therefore, an increase in DNA damage supports the increase in the IPR (or $L_{d\text{-}\mathrm{IPR}}$) value that measures the increase in the spatial structural disorder, or spatial disorder strength, of the spatial molecular mass density.

### 4.2 Plausible Reasons for an Opposite Change in the $\langle \mathrm{IPR}\rangle ∼L_{d\text{-}\mathrm{IPR}}$ of DNA and Histone Molecules

Fetal alcoholism or alcohol consumed by the fetus might have caused the histone protein modifications, which in turn could be responsible for enhancing the relaxation of chromatin, causing lower mass density fluctuations and hence a smaller ⟨IPR⟩ value. H3K27me3 antibody is the trimethylation of lysine 27 on a histone protein, and the addition of alcohol causes the loss of the H3K27me3 methylation process. This loss of methylation may have played a role in DNA unwinding and gene expression. This decrease in the ⟨IPR⟩ value or disorder strength is due to the loss of methylation from the loosely attached histones but not from the tightly attached histone to the DNA. Furthermore, fetal alcoholism could result in the loose holding of nucleosomes, which causes a more relaxed state of chromatin. The relaxed state of chromatin increases the homogeneity in histone, reducing the mass density fluctuation or refractive index fluctuation. Therefore, we can conclude that alcohol consumed by a pregnant mother is consumed by the fetus which results in the upregulation or downregulation in the expression of certain gene types in pup brain cell nuclei could have resulted in FAS disorder or other neurological abnormalities.

In summary, we probed the structural changes in tissue, and spatial molecular specific DNA and histone of mice pups’ brains, which are initially at the nanoscale level, exposed to fetal alcoholism. Preliminary results are promising, and this invites a detailed study to interconnect these results and explain how they are related to long-term brain abnormalities.
